# High *in Vitro* Anti-Tumor Efficacy of Dimeric Rituximab/Saporin-S6 Immunotoxin

**DOI:** 10.3390/toxins8060192

**Published:** 2016-06-21

**Authors:** Massimo Bortolotti, Andrea Bolognesi, Maria Giulia Battelli, Letizia Polito

**Affiliations:** Department of Experimental, Diagnostic and Specialty Medicine-DIMES, Alma Mater Studiorum, University of Bologna, Via San Giacomo 14, 40126 Bologna, Italy; massimo.bortolotti2@unibo.it (M.B.); mariagiulia.battelli@unibo.it (M.G.B.); letizia.polito@unibo.it (L.P.)

**Keywords:** B-cell lymphomas, immunotherapy, immunotoxin, ribosome-inactivating protein, Rituximab, Saporin-S6

## Abstract

The anti-CD20 mAb Rituximab has revolutionized lymphoma therapy, in spite of a number of unresponsive or relapsing patients. Immunotoxins, consisting of toxins coupled to antibodies, are being investigated for their potential ability to augment Rituximab efficacy. Here, we compare the anti-tumor effect of high- and low-molecular-weight Rituximab/saporin-S6 immunotoxins, named HMW-IT and LMW-IT, respectively. Saporin-S6 is a potent and stable plant enzyme belonging to ribosome-inactivating proteins that causes protein synthesis arrest and consequent cell death. Saporin-S6 was conjugated to Rituximab through an artificial disulfide bond. The inhibitory activity of HMW-IT and LMW-IT was evaluated on cell-free protein synthesis and in two CD20^+^ lymphoma cell lines, Raji and D430B. Two different conjugates were separated on the basis of their molecular weight and further characterized. Both HMW-IT (dimeric) and LMW-IT (monomeric) maintained a high level of enzymatic activity in a cell-free system. HMW-IT, thanks to a higher toxin payload and more efficient antigen capping, showed stronger *in vitro* anti-tumor efficacy than LMW-IT against lymphoma cells. Dimeric HMW-IT can be used for lymphoma therapy at least for *ex vivo* treatments. The possibility of using HMW-IT augments the yield in immunotoxin preparation and allows the targeting of antigens with low internalization rates.

## 1. Introduction

CD20 (B1) is a membrane protein highly expressed by mature B lymphocytes. This cluster determinant represents an excellent target for monoclonal antibody (mAb)-based immunotherapy because of several favorable properties: (i) it is expressed on approximately 90% of B-cell non-Hodgkin’s lymphomas (NHLs); (ii) it is not expressed on B cell precursors nor on other tissues; (iii) it is widely expressed on the cell membrane; and (iv) it is not normally shed from the cell [1]. Rituximab is an anti-CD20 mouse-human chimeric mAb that has proven to be effective for the treatment of CD20^+^ NHLs and chronic lymphocytic leukemia. This mAb can activate different cell death mechanisms, primarily complement-dependent cytotoxicity (CDC), but also antibody-dependent cell cytotoxicity (ADCC) and, to a lesser extent, apoptosis. Moreover, Rituximab can raise the T-cell response against malignant clones [2,3,4].

Rituximab was the first antibody approved by the US FDA for the treatment of recurrent/refractory follicular NHL. Since 1997, Rituximab as a single agent or in combination with chemotherapy has revolutionized lymphoma therapy. Unfortunately, Rituximab is not effective for all patients, and another problem arises from an acquired resistance to Rituximab that has been reported for some patients [5].

Therefore, various strategies have been prospected to achieve higher anti-tumor response and longer remission duration. For example, Rituximab efficacy has been augmented by conjugation to active moieties, such as radionuclides (radioimmunoconjugates) [6], toxic enzymes or lectins (immunotoxins, ITs) [7,8], and drugs (immunoconjugates) [9,10].

An increase in the efficacy of Rituximab against human B-cell lymphoma xenografts was obtained after conjugation to the anti-cancer drug calicheamicin in preclinical models with either nude or SCID mice [9]. Rituximab-conjugated to doxorubicin-loaded microbubbles, combined with ultrasound irradiation, were tested on Raji lymphoma cells. In this case, the increase in toxicity was encouraging but still modest [10].

Better results were achieved with radioimmunoconjugates, which were obtained by radiolabeling Rituximab and other anti-CD20 mAbs with ^131^I or ^90^Y, but, despite the good results reported in the preliminary studies, most treated patients relapsed [6].

The anti-tumor effect of mAbs may also be increased via the conjugation to a toxic molecule, thus obtaining chimeric proteins, called immunotoxins, in order to add cytotoxic properties to the specificity of the antibody. Both bacterial and plant toxins have been used to obtain immunotoxins [11,12,13,14]. Among plant toxins, the most utilized for conjugates are ribosome-inactivating proteins (RIPs), which can mainly be divided into the following two groups: type 1, consisting of a single-chain protein with enzymatic activity, and type 2, consisting of an enzymatic A-chain linked to a B-chain with lectin properties [15]. RIPs are a class of enzymes that is widely distributed in the plant kingdom. RIP activity was first identified as rRNA *N*-glycosylase (EC 3.2.2.22), which specifically removes the A4324 adenine residue inside the universally conserved GAGA sequence on the ricin/sarcin region of the 28S rRNA in the 60S subunit of the rat ribosome. The adenine removal impairs ribosomes in an irreversible way, resulting in the inhibition of protein synthesis [15]. RIPs also show *in vitro* *N*-glycosylase activity on other substrates, such as DNA, mRNA, tRNA, and poly(A) [16,17]. For this reason, the RIPs have been proposed to be classified as a polynucleotide: adenosine glycosylases [18,19].

Saporin-S6 is a potent and stable type-1 RIP that is often used to construct immunotoxins [7,13] and has also been tested in clinical trials [12]. The ability to act on different substrates makes saporin-S6 able to kill cells by triggering several mechanisms of death such as apoptosis and necroptosis [20], and autophagy and oxidative stress [21], as already reported for other RIPs [22]. Saporin-S6 has also been detected in the nucleus of intoxicated cells, and early DNA strand breaks were simultaneously observed, suggesting a direct action on nuclear DNA [23].

In a previous study, we conjugated Rituximab to saporin-S6 and evaluated its anti-tumor activity in NHL cells. This immunotoxin was extremely cytotoxic to target cells. The conjugate also induced apoptosis in 95% of treated cells at a concentration of 10 nM. The co-administration with the chemotherapy drug fludarabine increased the cytotoxicity of Rituximab/saporin-S6 and caused the complete elimination of the malignant population [24].

Further, Flavell and coworkers [25] showed that the combination of Rituximab + anti-CD19 BU12/Saporin-S6 immunotoxin had a strong *in vitro* cytotoxic effect on Ramos cell line. In SCID-Ramos mice, the combination of the immunotoxin and Rituximab led to the complete survival of all animals that were disease-free at day +120.

In this study, we compare the *in vitro* anti-tumor activity of two conjugates consisting of anti-CD20 Rituximab and saporin-S6, characterized by a different number of mAb and RIP molecules linked together.

## 2. Results

**Figure 1:**
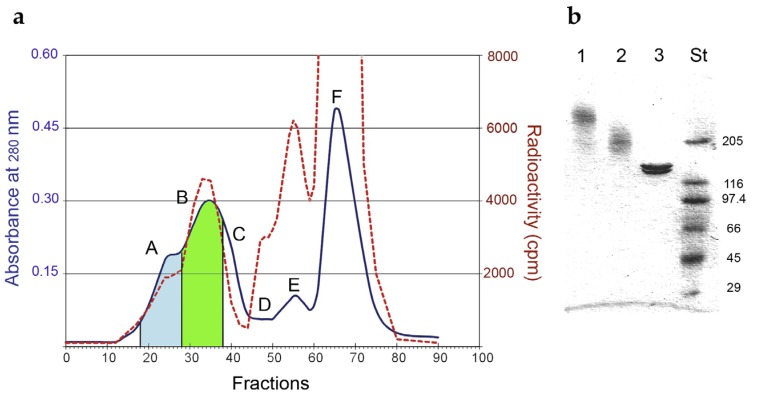
(**a**) Elution profile of gel-filtration chromatography on a Sephacryl S-200 HR column of Rituximab/saporin-S6 conjugate. The blue line represents the A_280_, and the red dashed line represents the radioactivity of the eluted fractions. The fractions corresponding to the peaks were pooled and indicated with capital letters: (A) high-molecular-weight immunotoxin (HMW-IT) (blue area); (B) low-molecular-weight immunotoxin (LMW-IT) (green area); (C) free Rituximab; (D) trimmers; (E) dimers and (F) monomers of saporin-S6. (**b**) Analysis of fractions corresponding to HMW-IT (1), LMW-IT (2), and unconjugated Rituximab (3) by SDS–PAGE under non-reducing conditions on a 4%–15% PhastGel. Standard molecular weights (St) are expressed in kDa.

The RIP saporin-S6 was conjugated to the anti-CD20 mAb Rituximab through an artificial disulfide bond. The optimal derivatization condition for Rituximab, to obtain a dimeric immunotoxin, was reached with 0.5 mM 2-iminothiolane, which yielded 3.66 thiol groups inserted *per* molecule. For saporin-S6 0.94 thiol groups *per* molecule were inserted using the linker 2-iminothiolane at a 1 mM concentration. The immunoconjugate was purified by gel filtration chromatography, and the fractions corresponding to the different peaks were pooled and analyzed by SDS–PAGE (Figure 1).

**Figure 2:**
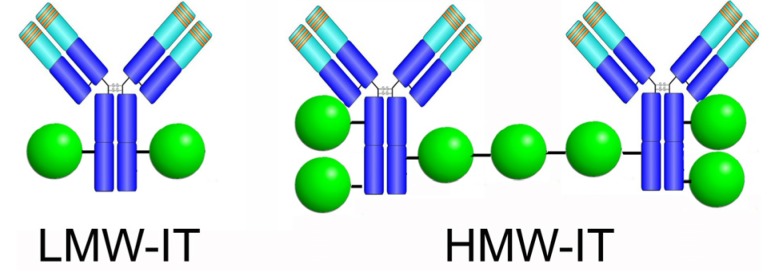
Possible structure of the most represented molecular species of LMW-IT and HMW-IT, generated from Rituximab chemical conjugation to saporin-S6.

We separated two different types of conjugates, a low-molecular-weight immunotoxin (LMW-IT) and high-molecular-weight immunotoxin (HMW-IT). The average molecular weight and the possible composition of the two conjugates were calculated on the basis of the elution volume, SDS-PAGE analysis, and RIP-to-antibody ratio, estimated by the ^125^I-RIP radioactivity and the A_280_. LMW-IT is a mixture of 210 kDa average molecular weight conjugates composed of one mAb and one or more RIP molecules. HMW-IT consists of a complex of two antibodies and more RIP molecules, with a 510 kDa average molecular weight (Figure 2).

The final yields of conjugated mAb and RIP were 53% and 9%, respectively (Table 1).

After conjugation, for both immunotoxins, we investigated whether saporin-S6 maintained its enzymatic activity, evaluating the inhibition of protein synthesis in a cell-free system (rabbit reticulocyte lysate). This test showed that protein synthesis was efficiently inhibited by both conjugates. The LMW-IT showed an IC_50_ of 7.31 × 10^−11^ M, which was comparable to that of native saporin-S6 (5.24 × 10^−11^ M). The HMW-IT showed an IC_50_ of 19.9 × 10^−11^ M, and this value was not significantly higher than that calculated for the LMW-IT (*p* = 0.082, as calculated by ANCOVA/Bonferroni test), demonstrating that HMW-IT also maintained very good cell-free protein synthesis inhibitory activity.

**Figure 3:**
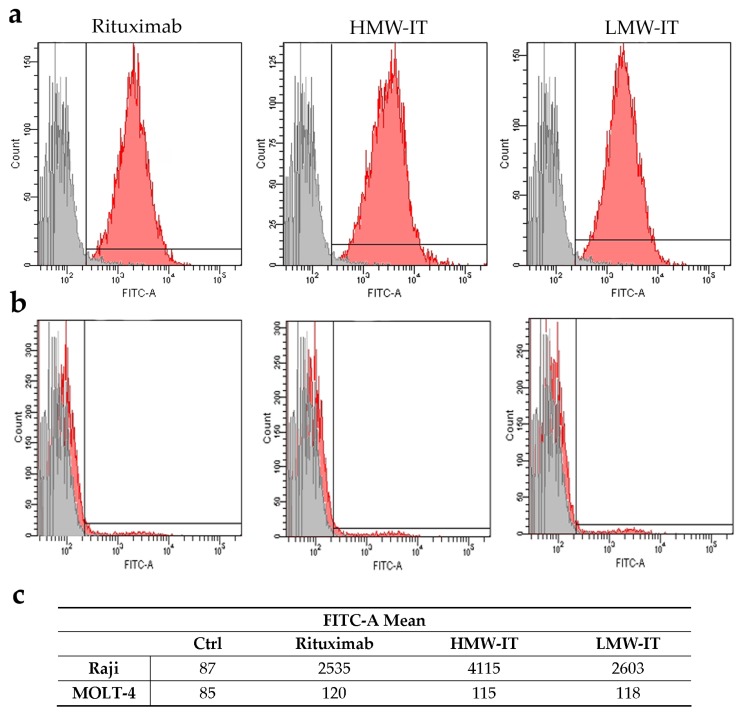
Cytofluorimetric analysis of Rituximab, HMW-IT, and LMW-IT binding on Raji (CD20^+^) (**a**) and MOLT-4 (CD20^−^) (**b**) cells. Grey histograms indicate the cells incubated without the mAb or immunotoxins. The fluorescence intensity, evaluated as FITC-A mean value, is also reported in the table (**c**).

By flow cytometry analysis, we demonstrated that the derivatization and conjugation procedures did not alter the mAb binding affinity. As compared to Rituximab, the LMW-IT maintained the same affinity for the CD20 antigen, while the HMW-IT showed an almost doubled antigen-binding property (Figure 3a). The specificity for the CD20 antigen of the immunotoxins, as well as of the free mAb, is demonstrated by the absence of binding to non-target MOLT-4 cells (CD20^−^) (Figure 3b).

**Figure 4:**
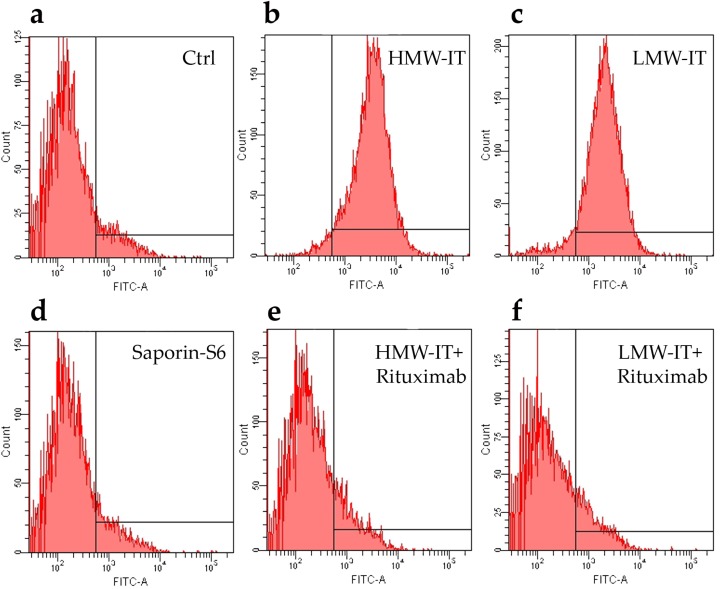
Immunospecificity of the immunotoxins towards CD20 expressing cells (Raji) by cytofluorometric analysis. The cells were incubated with HMW-IT and LMW-IT in the absence (**b**,**c**) or in the presence (**e**,**f**) of 100-fold molar excess of Rituximab. The immunotoxins binding was detected by rabbit antisera against saporin-S6, followed by an anti-rabbit FITC antibody. Control samples were run with complete medium alone (see Matherials and Methods) (**a**) or with saporin-S6 (**d**).

The binding of both HMW-IT and LMW-IT to the CD20 antigen, revealed by an anti-saporin-S6 antibody, was blocked by an excess of unconjugated Rituximab, thus indicating that the immunotoxins retained the immunospecificity and binding characteristics of Rituximab (Figure 4). No binding was observed with unconjugated saporin-S6.

**Figure 5:**
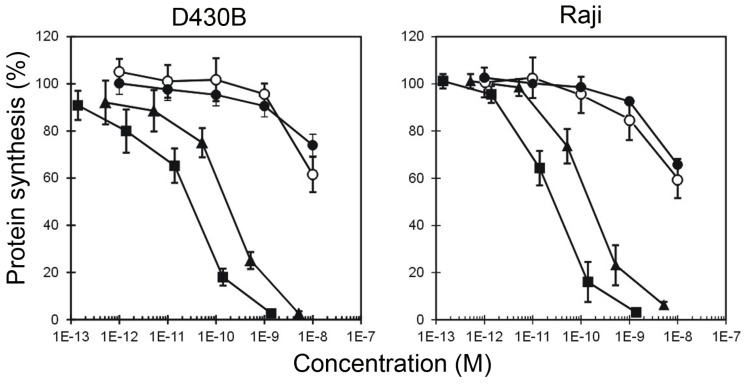
Cell protein synthesis inhibition assay. D430B and Raji cells were treated with HMW-IT (square), LMW-IT (triangle), free saporin-S6 (white circle) or a mixture of unconjugated Rituximab and saporin-S6 (black circle). The cells (5 × 10^3^) were seeded in 96-well plates in a total volume of 200 μL of complete medium (see Matherials and Methods) containing various concentrations of substances. After 96 h of incubation and a further 6 h with [^3^H]-leucine, the incorporated radioactivity was determined. The results are the means of two independent experiments, each performed in triplicate. SD was <10%. Data were analyzed by the ANCOVA/Bonferroni test. The inhibition of protein synthesis by HMW-IT differed significantly (*p* < 0.001) from that by LMW-IT. Both immunotoxins significantly inhibited protein synthesis with respect to the free RIP and mixture (*p* < 0.0001).

Cytotoxicity experiments were performed on two CD20^+^ lymphoma cell lines, namely D430B and Raji, by measuring protein synthesis inhibition after 96 h. After conjugation with Rituximab, saporin-S6 showed an increased cytotoxicity to target cells by 3 logs for HMW-IT and 2 logs for LMW-IT, with IC_50_ values of 10^−11^ M and 10^−10^ M, respectively (Figure 5, Table 2). The HMW-IT in both cell lines was significantly more active than LMW-IT. Protein synthesis was completely inhibited by HMW-IT at a 10^−9^ M concentration, whereas LMW-IT had the same effect at a 10^−8^ M concentration. Free saporin-S6 or a mixture of unconjugated Rituximab and saporin-S6 were able to significantly inhibit protein synthesis only at a 10^−8^ M concentration, and it was not possible to calculate an IC_50_ value. No inhibition was induced by free mAb.

The specificity for CD20 of the two immunotoxins was investigated with two non-target (CD20^−^) cell lines, MOLT-4 and Jurkat. In both cases, the cytotoxicity of HMW-IT and LMW-IT was not increased in comparison with the mixture of unconjugated Rituximab and saporin-S6, or to the RIP alone. In any case, IC_50_ values were higher than the maximum tested concentration (10^−8^ M) (Table 2).

**Figure 6:**
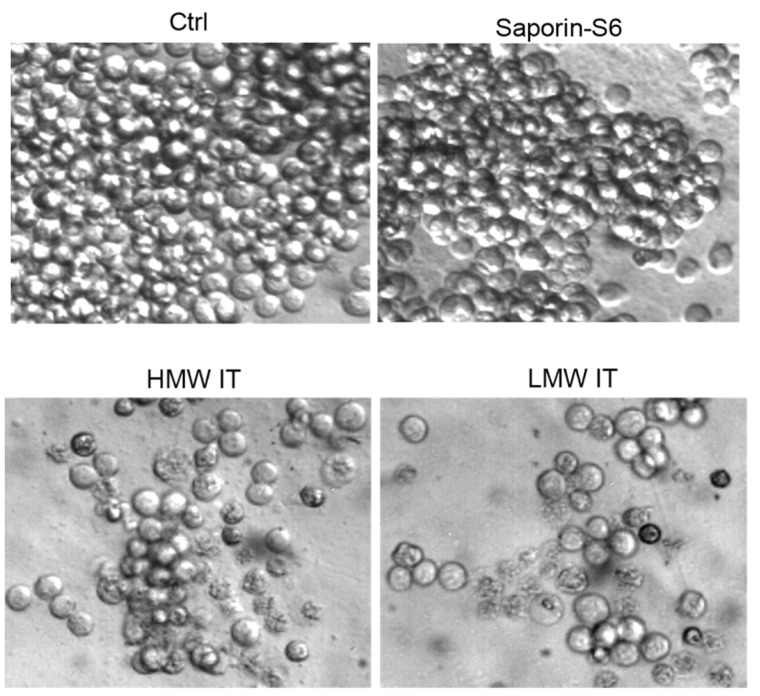
Morphology of Raji cells assessed by phase contrast microscopy. Cells were treated for 96 h with 10^−9^ M HMW-IT or LMW-IT or saporin-S6. Control cultures (Ctrl) were grown in the absence of immunotoxin or saporin-S6. Magnification: 400×.

The visual inspection of Raji cells treated with immunotoxins showed morphological features characteristic of apoptosis, including cell shrinkage, cell membrane budding, and cytoplasmic condensation (Figure 6). The morphology of Raji cells treated with saporin-S6 in the same conditions of immunotoxins did not differ from control cultures.

## 3. Discussion

In the last twenty years, thousands of lymphoma patients have benefited from Rituximab’s anti-tumor activity [26]. Despite the successes reported for Rituximab, some patients do not respond to the therapy, and others acquire resistance to Rituximab after the first series of treatments [5]. To explain the mechanisms of this resistance, different alterations in host immunologic factors have been assumed, such as (i) CDC unresponsiveness due to altered expression of CD46, CD55, and CD59 (complement-regulatory proteins) on tumor cells; (ii) ADCC resistance due to changes in the lipid raft and FcγRIIIa polymorphisms, causing failed recognition of the CD20/antibody complex by effector cells; (iii) selection of apoptosis resistant clones as a consequence of repeated exposure to Rituximab; and (iv) selection of CD20^−^ tumor clones [27,28].

In addition to CDC and ADCC, it has been reported that Rituximab can directly trigger cell death by apoptosis. However, a low percentage of CD20^+^ cells undergo apoptotic death after the binding of Rituximab to its target antigen [2,29]. To augment the antibody ability to induce apoptosis in CD20 cells, different strategies have been exploited, mainly involving the crosslinking of more CD20 receptors on the surface of lymphoma B cells [30].

An enhancement of the pro-apoptotic action of Rituximab was obtained by hypercross-linking Rituximab with anti-human or anti-mouse immunoglobulins [31]. Moreover, a two-step therapeutic approach has recently been described. This method is based on the administration of an anti-CD20 Fab’ antibody linked to a single-stranded oligonucleotide, followed by a complex consisting of multiple copies of the complementary oligonucleotide, linked to a linear polymer backbone. The consecutive administration of these drug-free components was proven to mediate CD20 crosslinking and to enhance apoptosis both *in vitro* and *in vivo* [32,33].

Ghetie and coworkers [34] proposed that CD20 capping could also be improved using Rituximab homodimers. They observed that homodimers, but not monomers, of Rituximab induced both apoptosis and necrosis of several B-lymphoma cell lines. Homodimers induced 33% apoptosis in the Ramos cell line, compared with 32% obtained with Rituximab monomers followed by goat anti-mouse Ig, and both values were significantly higher than that obtained with Rituximab monomers (6% of apoptotic cells). This enhanced effect can be attributed to the greater avidity of homodimers that causes both an increase in the cross-linking of adjacent CD20 antigens and a lower antigen dissociation of the complex. Moreover, *in vivo*, this one-step procedure is certainly simpler to apply than a two-step procedure.

Starting from the above observations, in this study, we compared the *in vitro* activity of two Rituximab/saporin-S6 immunotoxins, characterized by a different number of mAb and RIP molecules linked together. In previous studies, an ideal immunotoxin was considered to be constituted by an average of one molecule of antibody linked to one-two RIP molecules. This type of conjugate, characterized by two antigen binding sites, was defined here as LMW-IT.

Previous studies evaluated the efficacy of immunotoxins containing a variable number of RIP molecules on different cell targets [35,36,37]. These works reported that the number of inserted RIP molecules was correlated to increased immunotoxin cytotoxicity, although in some cases with a reduction of antigen binding capacity.

In this research, we investigated a different approach; in addition to the LMW-IT, we purified a HMW-IT that is characterized by four antigen-binding sites. Increasing the mAb and/or RIP number of thiol groups inserted, it was possible to obtain both the LMW-IT and the HMW-IT in the reaction mixture. The HMW-IT is characterized by a higher avidity for CD20 and a higher toxin payload compared with the LMW-IT. Accordingly, the cytofluorimetric analysis showed higher fluorescence intensity for HMW-IT- than for LMW-IT-binding to CD20^+^ Raji cells. Additionally, through flow cytometry analyses, we demonstrated that immunospecificity was retained for both types of construct. This was established by experiments of immunotoxin binding to target (CD20^+^) and non-target (CD20^−^) cells and by competition binding experiments with an excess of unconjugated Rituximab.

In cell-free experiments, the LMW-IT maintained an almost unaltered capability to inhibit protein synthesis in a cell-free system compared to saporin-S6. Under the same experimental conditions, the HMW-IT showed a higher, even if not significant, IC_50_ value, corresponding to lower enzymatic activity than LMW-IT. This loss of enzymatic activity for the HMW-IT is probably due to the fact that it is composed of a larger number of RIP molecules with two chemically inserted SH groups. A high derivatization degree of saporin-S6 leads to alterations of the three-dimensional toxin structure with consequent reduction of its affinity for the ribosomal substrate.

In cell experiments, both Rituximab/saporin-S6 conjugates showed a strong effect on the two CD20^+^ lymphoma-derived cell lines, causing a complete block of protein synthesis at a concentration significantly higher for LMW-IT than for HMW-IT. In addition, the immunospecificity of immunotoxins was demonstrated by the lack of cytotoxicity for CD20^−^ cell lines.

Though HMW-IT was less active than LMW-IT in cell-free experiments, in both cell lines it showed a stronger effect. This could be explained by the higher avidity toward the CD20 antigen (four antigen binding sites of the HMW-IT *vs* two of the LMW-IT) with a consequently better internalization efficiency that could balance the lower affinity of the toxic molecule for the ribosomal substrate.

In agreement with the results reported for Rituximab homodimers, HMW-IT (which can be considered a homodimer immunotoxin) showed a significant increase in anti-tumor activity. This indicates that the increased number of mAb *per* immunotoxin molecule leads to a more efficient internalization by enhancing CD20 capping. This, together with the higher toxic payload of HMW-IT, results in a large amount of toxin being delivered inside the cell, thus increasing the lethal effect.

## 4. Conclusions

Experiments carried out in our laboratory showed that the HMW-IT has better anti-tumoral features than the LMW-IT, suggesting a possible use in lymphoma therapy, at least for *ex vivo* treatments. The possibility of utilizing both high- and low-molecular-weight conjugates strongly augments the yield in the preparation of chemically formulated immunotoxins. Moreover, the potential of enhancing the endocytosis of the immunotoxin by using a dimeric HMW molecule opens up the possibility of also utilizing antigens with a low internalization rate. However, in view of a possible clinical use, LMW-IT might penetrate better than HMW-IT into the tumor mass because of the smaller size and would have greater access to the CD20 antigen on neoplastic cells. Further experiments in animal models are necessary to establish the possible therapeutic value of the HMW-ITs.

## 5. Materials and Methods

### 5.1. Immunotoxin Preparation

Rituximab (anti-human CD20 mAb) was purchased from Roche (Milan, Italy). The type-1 RIP saporin-S6, from the seeds of *Saponaria officinalis*, was purified as previously described [38]. Saporin-S6 was labeled with ^125^I using the Iodogen reagent (Pierce Chemical Co., Catex, Dallas, TX, USA) according to the manufacturer’s instructions.

The RIP and the mAb were chemically linked through a disulfide bond between the inserted thiol groups, as already described [39,40]. Briefly, Rituximab and saporin-S6, containing a trace of ^125^I-RIP, were modified by adding 2-iminothiolane. Thiol groups were inserted into each molecule by an imidoester reaction between 2-iminothiolane and the primary amino groups of the proteins. The derivatized RIP was reduced with 20 mM 2-mercaptoethanol, filtered through a Sephadex G25 column (GE-Healthcare, Buckinghamshire, UK), and then mixed with the derivatized mAb, in a 10:1 molar ratio. The mixture was allowed to react for 16 h at room temperature. The resulting high-molecular-weight immunotoxin (HMW-IT) and low-molecular-weight immunotoxin (LMW-IT) were separated from the unreacted reagents and from saporin-S6 homopolymers by gel filtration on a Sephacryl S200 high-resolution column (100 cm × 2.5 cm) (GE-Healthcare), equilibrated and eluted with phosphate-buffered saline (PBS, 0.14 M sodium chloride in 5 mM sodium phosphate buffer, pH 7.4) [24].

The immunotoxins were analyzed by SDS-PAGE under non-reducing conditions. Proteins were incubated in sample buffer (40 mM Tris-HCl pH 6.8, 2% SDS, 0.005% bromophenol blue), containing 1 mg/mL iodoacetamide, for 30 min at room temperature, analyzed on a 4%–15% PhastGel gradient (GE-Healthcare), and then stained with Coomassie brilliant blue, as described in [24].

The RIP-to-antibody ratio in the conjugates was estimated from the ^125^I-RIP radioactivity and from the protein concentration calculated from the A_280_. The radioactivity enabled the calculation of the saporin-S6 contribution to the A_280_ of the conjugate. By subtracting this amount from the total absorbance of each immunotoxin, the Rituximab contribution to the A_280_ of the conjugates was calculated. Then, the absorbance of each protein was divided for the respective molar extinction coefficient to obtain the relative concentration of RIP and mAb in the two immunotoxins [41].

### 5.2. Cell Lines

The activity of the immunotoxins was assayed on the CD20^+^ cell lines Raji, derived from a Burkitt’s lymphoma (ATCC number CCL-86™, and D430B, an Epstein Barr virus infected B cell line [42]. The CD20^−^ MOLT-4 (ATCC number CRL-1582™) and Jurkat (ATCC number TIB-152™) cell lines were used as non-target cells. All the cell lines were from long-term culture of our department.

Cells were maintained in RPMI 1640 medium, supplemented with 10% heat-inactivated fetal bovine serum (FBS), 2 mM l-glutamine, 100 U/mL penicillin, and 100 μg/mL streptomycin, (hereafter named complete medium), in humidified air with 5% CO_2_ at 37 °C. Viability was checked before each experiment by trypan blue dye exclusion. All medium and reagents for cell cultures were purchased from Sigma (Sigma-Aldrich, St. Louis, MO, USA).

### 5.3. Protein Synthesis Inhibition Assays

The inhibitory activity of the immunotoxins on cell-free protein synthesis was evaluated with a rabbit reticulocyte lysate as described in [43]. Immunotoxins were previously reduced with 20 mM 2-mercaptoethanol for 30 min at 37 °C, appropriately diluted, and then added to a reaction mixture. Each experiment was carried out in duplicate. The concentration of immunotoxin, expressed as RIP content, causing 50% inhibition of leucine incorporation (IC_50_) was calculated by linear regression analysis.

The cytotoxicity of the immunotoxin was evaluated from the inhibition of cellular ^3^H-leucine incorporation. Cells (5 × 10^3^/well) were seeded in 96-well microtiter plates (Falcon, Becton Dickinson, Franklin Lakes, NJ, USA) in 100 μL of complete medium, and 100 μL of Rituximab-containing immunotoxin was added to final concentrations ranging from 10^−13^ to 10^−8^ M. Concentrations are expressed as immunotoxin content. Control samples were run with RIP alone. After 96 h, cells were washed, 5 kBq of l-[4,5-^3^H]leucine (Amersham) was added, and after a further 6 h, cells were harvested with an automatic cell harvester (Skatron Instruments, Lier, Norway) onto glass-fiber diskettes. The radioactivity incorporated was determined as previously described [44,45].

The morphological analysis of the treated cells was conducted through phase-contrast microscopy directly in 96-well plates using a digital camera from Motic Microscopes (Xiamen, Fujian, China). The presence of cellular morphological changes was examined in Raji cells treated for 96 h with 10^−9^ M HMW-IT and LMW-IT.

### 5.4. CD20 Affinity Cytofluorimetric Experiments

Rituximab affinity for CD20 antigen was evaluated by indirect immunofluorescence. Cells (5 × 10^5^ in complete medium) were treated with Rituximab or immunotoxins, at the final concentration of 10^−8^ M as described in [24]. After cell fixation with 70% ethanol, the CD20 binding of Rituximab, HMW-IT, and LMW-IT was assessed through flow cytometry on a FACSAria BD Analyzer using FACSDiva software (Becton, Dickinson and Company, FranklinLakes, NJ, USA). The intensity of fluorescence, evaluated as FITC-A mean value, was used as the measure of the binding capability.

In the Rituximab competition experiments, cells (5 × 10^5^ in complete medium) were collected into round-bottom tubes at 4 °C and incubated with 50 μL of Rituximab at 10^−6^ M concentration or with 50 μL of PBS, 1% FCS. After 30 min, cells were centrifuged at 500× *g* for 5 min at 4 °C and treated for 30 min at 4 °C with free saporin-S6, HMW-IT or LMW-IT, at a final concentration of 10^−8^ M. Control samples were run with complete medium alone. After a wash in PBS containing 1% FCS, cells were incubated for 30 min at 4 °C with 50 μL of rabbit antisera against saporin-S6 (1:100). After a further wash, 50 μL of anti-rabbit FITC antibody were added for 30 min at 4 °C. After three washes in cold PBS, 1% FCS, the samples were fixed with 70% cold ethanol. The fluorescence intensity was assessed as above described.

### 5.5. Statistical Analyses

The statistical analyses for the *in vitro* experiments were conducted using XLSTAT-Pro software, version 6.1.9 (Addinsoft, New York, NY, USA, 2003). The results are given as the means ± S.D. The data were analyzed using ANCOVA and ANOVA tests, with Bonferroni’s correction for multiple comparisons.

## Figures and Tables

**Table 1:** Characteristics of low- and high-molecular-weight Rituximab/saporin-S6 immunotoxins.

Cnjugates	RIP/mAb Molar Ratio	Average Mw (kDa)	IC_50_^1^ (M × 10^−11^)	Yield (%) ^2^	
				mAb	RIP
**LMW**	1.93	210	7.31	37	5
**HMW**	3.60	510	19.9	16	4

^1^ Concentration causing 50% inhibition of protein synthesis in a cell-free system (rabbit reticulocyte lysate). The IC_50_ of native saporin-S6 is 5.24 × 10^−11^ M. ^2^ Amount of mAb and ribosome-inactivating protein (RIP) recovered in the conjugates, each expressed as a percentage of the starting protein amount.

**Table 2:** Effects of HMW-IT and LMW-IT on cell protein synthesis.

Tested substances	CD20^+^ Target Cells		CD20^−^ not Target Cells	
	D430B	Raji	MOLT-4	Jurkat
	IC_50_ (M) ^1^			
HMW-IT	2.93 × 10^−11^	2.26 × 10^−11^	>10^−8^	>10^−8^
LMW-IT	1.98 × 10^−10^	1.52 × 10^−10^	>10^−8^	>10^−8^
Saporin-S6	>10^−8^	>10^−8^	>10^−8^	>10^−8^
Saporin-S6 + Rituximab	>10^−8^	>10^−8^	>10^−8^	>10^−8^

^1^ The IC_50_ of the immunotoxins refers to the RIP content.

## Abbreviations

The following abbreviations are used in this manuscript:

mAb	monoclonal antibody
NHL	non-Hodgkin’s lymphoma
CDC	complement-dependent cytotoxicity
ADCC	antibody-dependent cellular cytotoxicity
RIP	ribosome-inactivating protein
HMW-IT	high-molecular-weight immunotoxin
LMW-IT	low-molecular-weight immunotoxin
PBS	phosphate-buffered saline
FBS	fetal bovine serum
